# Investigation of Diffusion Induced Fiber–Matrix Interface Damages in Adhesively Bonded Polymer Composites

**DOI:** 10.3390/polym17121672

**Published:** 2025-06-17

**Authors:** Dudu Mertgenç Yoldaş

**Affiliations:** Department of Mechanical and Metal Technologies, Dokuz Eylul University Izmir Vocational School, Buca, 35360 Izmir, Turkey; dudu.yoldas@deu.edu.tr

**Keywords:** adhesive bonding, composite materials, diffusion, durability, interfacial damage, three point bending test

## Abstract

Composite materials have the advantages of high strength and low weight, and are therefore used in many areas. However, in humid and marine environments, mechanical properties may deteriorate due to moisture diffusion, especially in glass fiber reinforced polymers (GFRP) and carbon fiber reinforced polymers (CFRP). This study investigated the damage formation and changes in mechanical properties of single-layer adhesive-bonded GFRP and CFRP connections under the effect of sea water. In the experiment, 0/90 orientation, twill-woven GFRP (7 ply) and CFRP (8 ply) plates were produced as prepreg using the hand lay-up method in accordance with ASTM D5868-01 standard. CNC Router was used to cut 36 samples were cut from the plates produced for the experiments. The samples were kept in sea water taken from the Aegean Sea, at 3.3–3.7% salinity and 23.5 °C temperature, for 1, 2, 3, 6, and 15 months. Moisture absorption was monitored by periodic weighings; then, the connections were subjected to three-point bending tests according to the ASTM D790 standard. The damages were analyzed microscopically with SEM (ZEISS GEMINI SEM 560). As a result of 15 months of seawater storage, moisture absorption reached 4.83% in GFRP and 0.96% in CFRP. According to the three-point bending tests, the Young modulus of GFRP connections decreased by 25.23% compared to dry samples; this decrease was 11.13% in CFRP. Moisture diffusion and retention behavior were analyzed according to Fick’s laws, and the moisture transfer mechanism of single-lap adhesively bonded composites under the effect of seawater was evaluated.

## 1. Introduction 

Composite materials are artificially created materials that are generally produced by combining at least two physically and/or chemically different phases in a certain arrangement; natural materials (e.g., wood) are excluded from this definition. There is a distinct interface between these phases, and the resulting composite offers properties that the components alone cannot provide [1]. Effective use of these materials, which offer durability and design flexibility, requires good knowledge of their mechanical behavior [2]. The behavior of the material, especially under variable external effects, such as cyclic loading, impact, temperature change and vibration, may vary depending not only on the properties of the components but also on many factors, such as interface quality, fiber–matrix interaction, fiber orientation and manufacturing processes [3]. In composite materials, the interface is a critical region where load transfer occurs between the matrix and fiber phases. Ensuring strain compatibility in this region is of great importance for the effective transfer of load from the matrix to the fibers. Any disruption at the interface eliminates the deformation compatibility between the matrix and fibers and prevents load transfer. Therefore, the structural integrity of composite materials depends largely on the stress compatibility at the interface [4]. In fiber-reinforced polymer (FRP) composites in particular, the strength of the material is determined not only by the mechanical properties of the components but also by the interfacial forces. In addition, the arrangement of the fibers with respect to the loading direction (fiber orientation) has a direct impact on the load-carrying capacity by determining the effective cross-sectional area of the load-carrying fibers [5]. Composite materials are widely preferred in many different application areas in the maritime sector, such as marine vessels, superstructures and commercial ships. However, the long-term and safe use of these materials in the marine environment depends on a good understanding of the effects of environmental factors on the material. In the marine environment, composites are exposed to various physical and chemical effects, such as absorption of sea water, ambient temperature, changes in pH levels, ultraviolet (UV) radiation and hydrostatic pressure. These factors can adversely affect both the structural properties and service life of composite materials [6]. Seawater absorption, in particular, causes the polymer matrix to absorb water, leading to volume increase and plasticization. This causes physical deterioration. Increased ambient temperature can cause stress accumulation within the material by increasing thermal expansion. Changes in pH levels, especially in acidic or basic environments, cause chemical deterioration in the matrix phase, which triggers processes such as hydrolysis. UV radiation can cause photodegradation in the polymer structure, resulting in effects such as color change, cracking and surface damage. The pressure effect creates stress on the microstructure, causing cracks to form and weakening of the fiber–matrix interfaces [7]. These effects can accumulate over time, leading to deterioration of the compatibility between the matrix and the fiber, separation at the interface, and overall serious losses in the mechanical performance of the composite material [8]. Moisture diffusion in polymers is of Fickian and non-Fickian nature. Viscoelastic structure and cracks lead to non-Fickian diffusion. Water diffusion depends on many factors such as resin, temperature, humidity, additives and fiber orientation. Moisture absorption is due to resin, while fibers absorb very little moisture. Carbon fibers are more resistant to moisture than glass [9,10]. Water penetration in composites is caused by matrix swelling, bond weakening, microcracks, osmotic effects and polymer-solvent interactions [11].

Some studies addressing this topic are discussed in the following paragraph.

Öner et al. (2017) [12] investigated the mechanical and thermomechanical properties of glass fiber reinforced composites prepared by adding carbon nanotubes (CNTs) to epoxy resin at 0.5–1.25% by weight. In the tensile test, the highest elasticity modulus and tensile strength were obtained with 0.5% CNT addition (15% and 46% increase). They observed a decrease due to agglomeration at 1% and 1.25% CNT rates. In the bending tests, the maximum flexural modulus (36% increase) was obtained with 0.75% CNT addition, and the highest flexural strength was obtained with 0.75% (23% increase). There was a decrease due to inhomogeneous mixing at 1%. According to Dynamic Mechanical Analysis (DMA), CNT addition changed the glass transition temperature; the highest Tg value was measured in samples with 0.5% addition. As a result, carbon nanotube reinforcement improves the tensile, flexural and thermomechanical properties of the composite material when used at appropriate rates.

Oner et al. (2018) [13] investigated tensile and flexural tests revealed that addition of nanoclay to epoxy matrix composites had a positive effect on the mechanical properties and the most significant improvements were observed at 0.75 wt% nanoclay content. At this concentration, tensile strength increased by about 6% (from 550 MPa to 582 MPa), and Young modulus improved by about 2% (from 66.991 MPa to 68.066 MPa), which was probably due to better dispersion and interaction between nanoclay and epoxy matrix. Similarly, flexural strength increased by about 6% (from 907 MPa to 957 MPa) and flexural modulus increased by 3% (from 57.610 MPa to 59.404 MPa). Improvements at 0.5 wt% nanoclay were minimal and a slight decrease in Young modulus was observed. However, in 1.25 wt% nanoclay, both tensile and flexural properties decreased, and this was attributed to poor dispersion and agglomeration in the nanoclay.

Mansouri et al. (2019) [14] investigated the hygrothermal aging effect of short fiber/woven laminates made of E-glass fiber and unsaturated polyester resin in salt and fresh water at 40 °C for one year. Gel-coated and uncoated samples were subjected to three-point bending tests. In the first month, a 37% decrease in elastic modulus and a 22% decrease in tensile strength were observed in distilled water. After the second month, the elastic modulus decreased by 24% in seawater and 14% in distilled water. It was stated that the deterioration in mechanical properties accelerated over time, and this was related to the increasing penetration of water. In addition, it was determined that the water absorption rates were similar in both environments and the absorption curve progressed rapidly at first, and then slowly.

Vizentin et al. (2022) [15] investigated Epoxy/glass and polyester/glass composites with different fiber arrangements were aged under sea for 6, 12 and 24 months to determine their mass change, microbial growth, tensile strength and morphology. Mass increase and tensile strength loss were observed in all samples due to seawater absorption and microorganism effect. While the strength loss was more pronounced in epoxy/glass samples, the most durable structure in polyester/glass samples was (0/45/90)s configuration. Microscopic analyzes revealed salt crystals and microorganism-induced deterioration on the matrix.

Mourad et al. (2010) [16] investigated the durability of glass/epoxy and glass/polyurethane composites under the effect of seawater and temperature. Immersion tests lasting from 3 months to 1 year were carried out at room temperature and 65 °C conditions. While seawater absorption increased with time and temperature, the matrix partially protected the fibers against abrasive effects. However, humidity caused deterioration by mechanisms such as swelling and weakening of interface bonds. Glass/polyurethane composites showed a 19% loss of tensile strength at room temperature and 31% at 65 °C over one year. No significant change was observed in the tensile strength of glass/epoxy. In addition, high temperature accelerated the deterioration by causing brittleness in glass/polyurethane composites.

Yalçınkaya et al. (2025) [17] investigated 0/90 oriented eight-layer GFRP and CFRP composites that were produced by hand lay-up and hot pressing methods. The samples were kept in seawater at 22.43 °C for 30 and 60 days. Charpy impact and three-point bending tests were applied before and after aging. After 30 days, deformation in bending tests was measured as 1.41% in GFRP and 0.31% in CFRP. The modulus of elasticity decreased by 7.48% and 7.46% in CFRP and 7.015% and 11.53% in GFRP. These results show that CFRP is more resistant to sea water than GFRP.

Vaishakh et al. (2024) [18] investigated the effect of moisture on moisture-induced unidirectional carbon-epoxy composites. Hygroscopic aging caused the formation of micro-voids at the fiber–matrix interface by water uptake. A sigmoidal function was used to model water uptake; based on the relationship between porosity and water content, aged composites were modeled as porous microstructures by computational micromechanics.

Zhang et al. (2025) [19] investigated the degradation mechanism of CFRP (carbon fiber reinforced polymer) in aqueous media and quantitatively analyzed the fiber–matrix interface degradation. A three-stage water absorption mechanism was identified; its steps included capillary flow, diffusion into the matrix, and interfacial debonding. Mechanical tests showed that matrix degradation caused reversible aging, while interfacial debonding caused irreversible aging. A proportional relationship was found between water content and mechanical performance degradation, and physically based models were developed to explain the aging processes.

Silva et al. (2025) [20] investigated the mechanical degradation of glass fiber/epoxy composites aged in salty environments. Composites using DGEBA epoxy and E-glass fabric were subjected to accelerated hygrothermal aging in salt spray chambers at 35, 55 and 70 °C. In the three-point bending test, mechanical properties changed with temperature. While the flexural strength decreased more at high temperatures, no significant change was observed in the flexural modulus due to its fiber-oriented structure.

Rodríguez-Dopico et al. (2024) [21] investigated the behavior of the bond between marine steel and bulk adhesive under the influence of seawater. Thermal properties were determined by TGA, and mechanical properties were evaluated by single-lap bonding and tensile tests. Seawater absorption was investigated by the gravimetric method, and DSC, FTIR and mechanical tests were applied before and after aging. It was found that water absorption obeyed Fick’s law, absorption accelerated under constant load and strength decreased. While the glass transition temperature increased in unloaded samples, no significant change was observed under load. Water content decreased over time in samples under constant load, which was attributed to more than one diffusion process.

Atakok et al. (2024) [22] investigated the seawater effect of GFRP and CFRP composites. They produced 0/90 oriented, plain weave seven-layer GFRP and eight-layer CFRP composites using the hand lay-up method. The samples were stored in both dry environments and seawater at a certain temperature for 1, 2 and 3 months. First, the moisture retention rates were measured, and then the samples were subjected to axial impact and three-point bending tests. The results show that, as the storage time of GFRP samples in seawater increased, the moisture retention rate and the energy absorbed in the impact tests increased, but the Young’s modulus decreased.

In this study, single-lap bonded joints made of 7-layer GFRP and 8-layer CFRP composites with a single layer of adhesive were immersed in separate containers filled with seawater taken from the Aegean Sea (salinity: 3.3–3.7%, temperature, 23.5 °C) for different durations of 30 days (720 h), 60 days (1440 h), 90 days (2160 h), 180 days (4320 h) and 450 days (10,800 h). After each soaking period, moisture absorption (%) of the samples was measured, and then three-point bending tests were applied. The damage in the materials was examined in detail by scanning electron microscope (SEM). The moisture absorption behavior of the joint samples in seawater environment was analyzed according to Fick’s diffusion laws. The diffusion process and moisture retention tendencies were evaluated comparatively, thus the moisture transfer mechanism in the samples exposed to seawater was understood. The results in this study showed when the damage at the bond and adhesive interface began, and at what stage seawater began to affect the mechanical properties of the composite material.

## 2. Materials and Methods

GFRP and CFRP composite materials were produced with different layer counts, fiber types and areal densities, featuring a 0/90 orientation and twill weave structure. An epoxy-based resin system was used, and prepreg production was carried out using a drum-type prepreg machine (Fibermak, İzmir, Türkiye). The production properties of GFRP and CFRP samples are presented in detail in Table 1.

**Figure 1:**
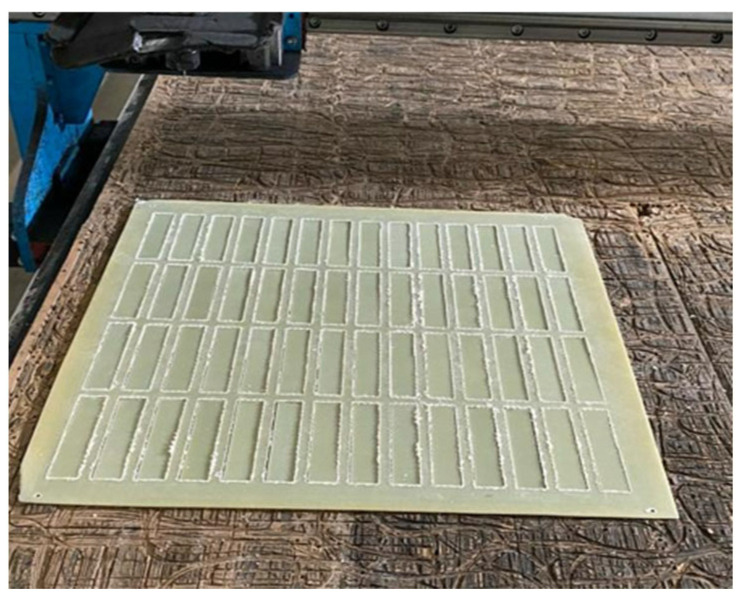
Cutting GFRP Samples Using CNC Router.

**Figure 2:**
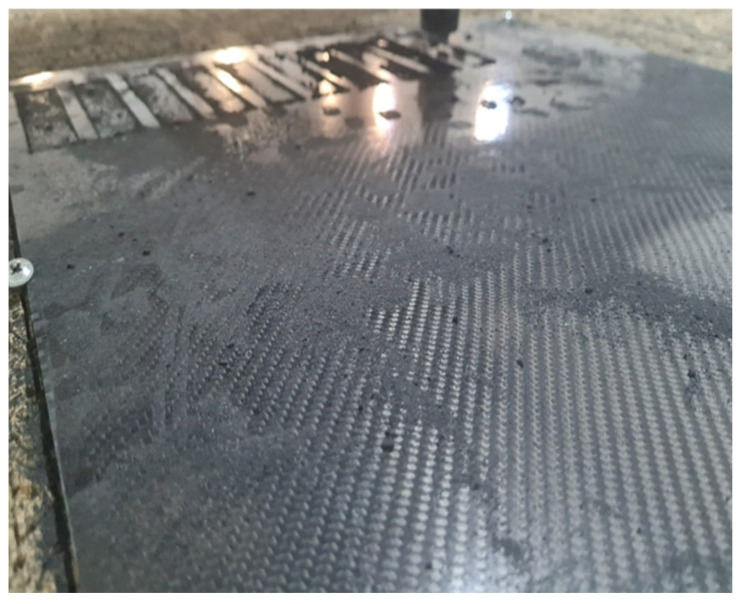
Cutting CFRP Samples Using CNC Router.

The prepreg material was kept for one day after the epoxy and hardener mixture was applied using the hand lay-up method. After the gelation process was completed, the composite production was carried out using the hot press method. As part of the production process, the material was cured for 1 h under 8–10 bar pressure at 120 °C (Table 2 and Table 3). The dimensions of the obtained GFRP and CFRP composite plates were determined as 500 mm × 500 mm and cut using a CNC Router (Fibermak, İzmir, Türkiye) (Figure 1 and Figure 2).

**Figure 3:**
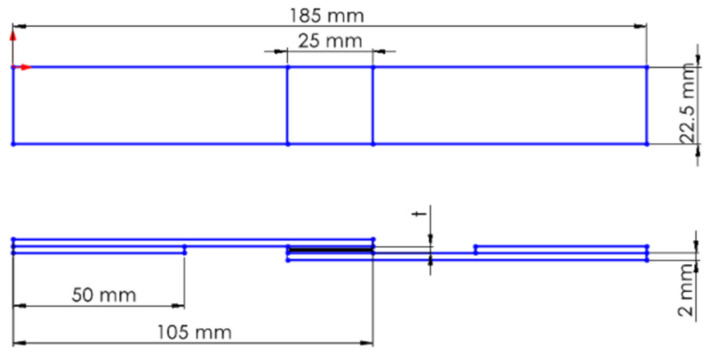
GFRP and CFRP schematic view of the test specimen.

As shown in Figure 3, GFRP and CFRP specimens were prepared by cutting according to the ASTM D5868-01 [23] standard.

**Figure 4:**
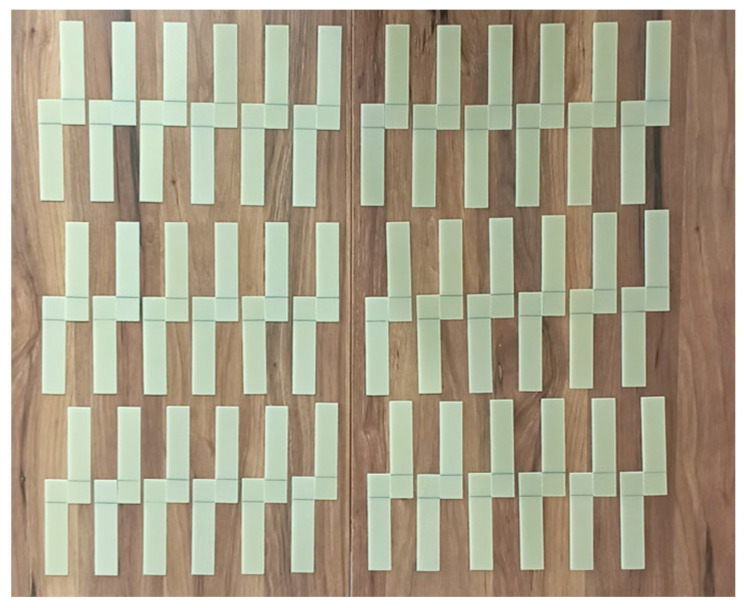
Marking of GFRP samples before bonding.

**Figure 5:**
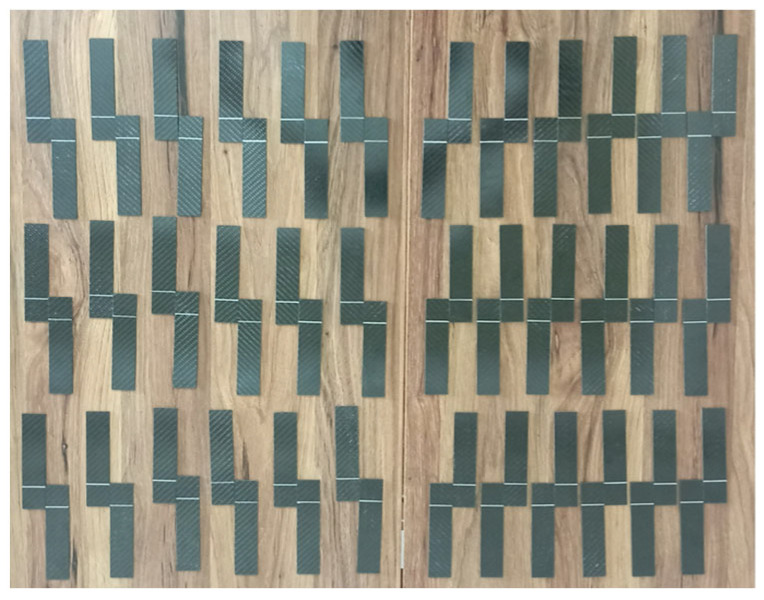
Marking of CFRP samples before bonding.

Before starting the bonding process, 25 mm lengths were measured and marked from the end of each of the GFRP samples in Figure 4 and CFRP samples in Figure 5.

**Figure 6:**
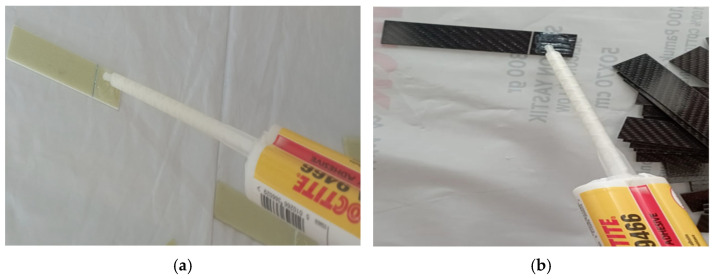
Application of Loctite Hysol-9466 adhesive on GFRP (**a**) and CFRP (**b**) samples.

The samples were prepared by bonding solvent-cleaned surfaces using the adhesive bonding method. The bonding process was carried out with two-component Loctite Hysol-9466 (Alpanhidrolik, Eskisehir, Turkey) epoxy adhesive, which was cured at room temperature and mixed in the application gun at a ratio of 2:1 (Figure 6).

**Figure 7:**
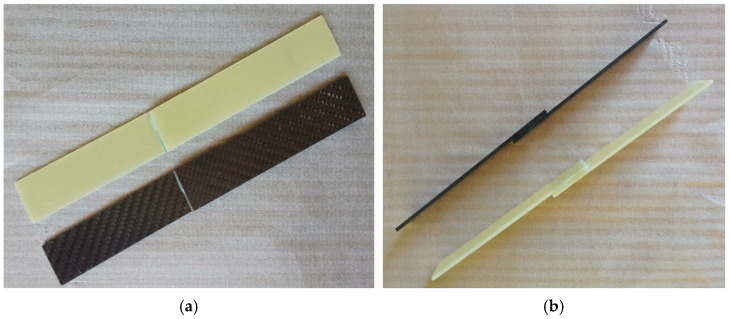
Front view (**a**) and left side view (**b**) of Loctite Hysol-9466 adhesive on GFRP and CFRP samples.

The literature indicates that adhesive layers of 0.1–0.3 mm thickness provide optimum bond strength, but thicknesses exceeding 0.6 mm reduce bond strength. This is explained by the fact that thin layers use the mechanical properties of the adhesive more effectively [24]. In accordance with the product data sheet, samples were tested after 7 days of curing at room conditions (Figure 7).

**Figure 8:**
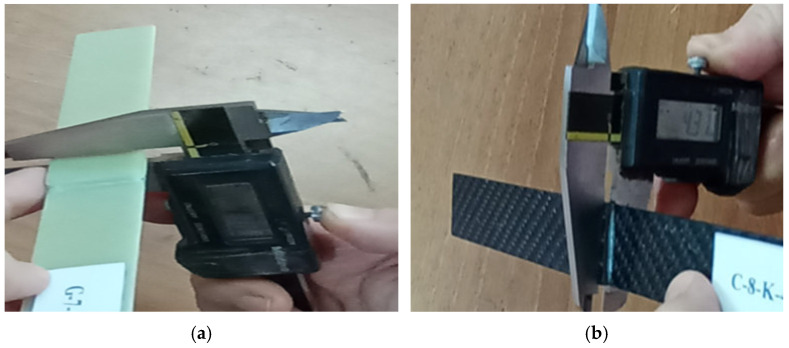
Thickness control of GFRP (**a**) and CFRP (**b**) bonded samples with digital caliper.

The evenness of the adhesive layer was checked with a digital caliper in the application made with a constant pressure of 0.1 MPa and a thickness of 0.2 mm (Figure 8).

GFRP and CFRP samples are coded to avoid confusion (Table 4).

For example, the sample coded G-7-K-1 is sample number 1, made of glass fiber, 7-layered, dry environment (not kept in sea water). The sample coded G-7-1A-1 is sample number 1, made of glass fiber, 7-layered, kept in sea water for 1 month (720 h).

**Figure 9:**
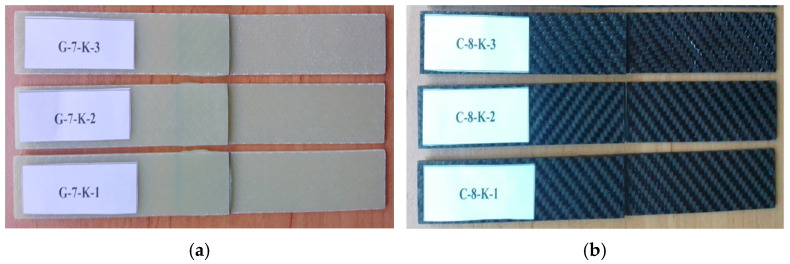
GFRP (**a**) and CFRP (**b**) coded specimen samples.

Meanwhile, the sample coded C-8-K-1 is sample number 1, made of carbon fiber, 8-layered, dry environment (not kept in sea water). The sample coded C-8-2A-1 is sample number 1, made of carbon fiber, 8-layered, kept in sea water for 2 months (1440 h) (Figure 9).

**Figure 10:**
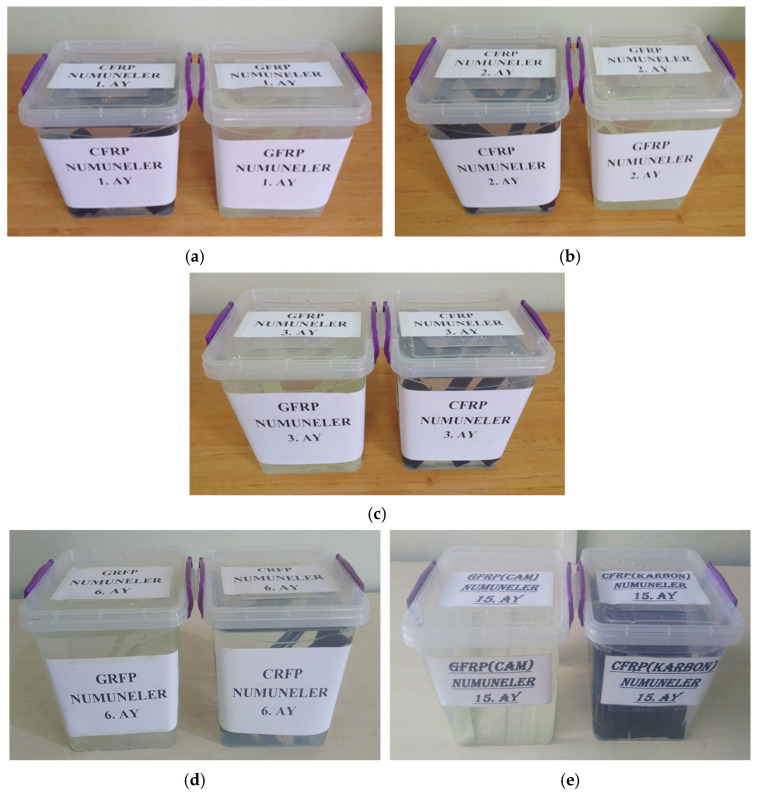
Soaking of GFRP and CFRP samples in sea water for (**a**) 1 month (720 h), (**b**) 2 months (1440 h), (**c**) 3 months (2160 h), (**d**) 6 months (4320 h) and (**e**) 15 months (10,800 h).

The samples were prepared with single overlap bonding and divided into 6 groups to be stored in dry conditions and in sea water for 1 month (720 h), 2 months (1440 h), 3 months (2160 h), 6 months (4320 h) and 15 months (10,800 h). The samples were first stored in dry conditions and then in seawater at 3.3–3.7% salinity and 23.5 °C for 1, 2, 3, 6 and 15 months, respectively. Seawater with the same salinity and constant temperature was used in each experiment (Figure 10).

### 2.1. Moisture Retention Performance of GFRP and CFRP Composites in Seawater Exposure

**Figure 11:**
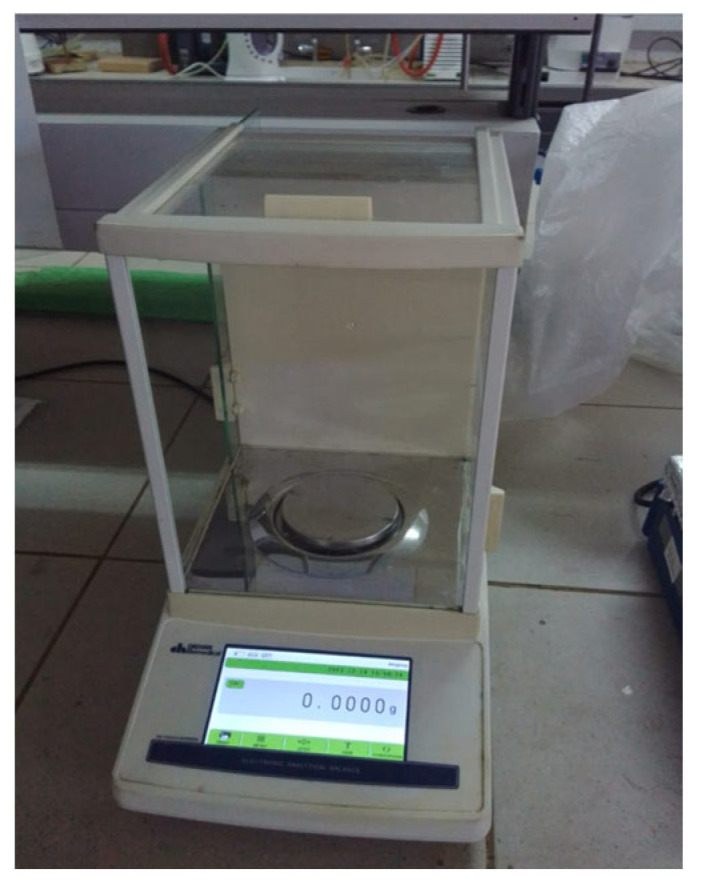
Daihan Biomedical precision scale.

GFRP and CFRP samples were prepared by single lap adhesive bonding method and aged in seawater conditions. First of all, in order to determine the moisture absorption, GFRP and CFRP samples were kept in separate containers in seawater with a salinity ranging from 3.3% to 3.7% at a temperature of 23.5 °C for 30 days (720 h), 60 days (1440 h), 90 days (2160 h), 180 days (4320 h) and 450 days (10,800 h). Weight measurements were made on a precision balance with 0.1 mg sensitivity in the Laboratory of Chemistry Department of DEU Faculty of Science, Daihan Biomedical (Daihan Biomedical, DAIHAN Scientific Lab & Medical Instruments Manufacturing, Seoul, Republic of Korea). For the sample type, measurements were made from three standard samples in seawater, and a total of 36 measurements were made. The average weight change graphs of the samples were created from the obtained data (Figure 11).

### 2.2. Three Point Bending Test

**Figure 12:**
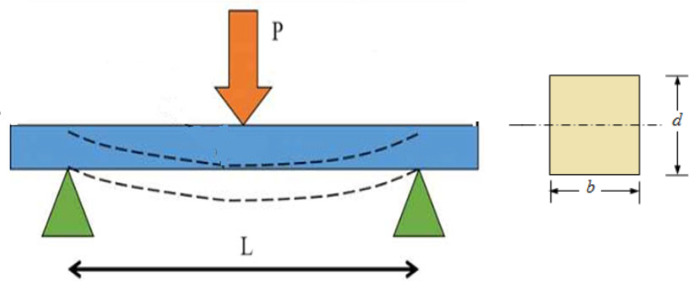
Schematic view of three-point bending test.

This test is performed to evaluate the mechanical properties of materials such as ductility, fracture strength and resistance to fracture. The main parameters measured in this test are bending strength (maximum stress value), flexural modulus (resistance to elastic deformation), yield strength (onset of plastic deformation), ductility and fracture toughness. In the three-point bending test, the material is supported from two ends, and the deformation is observed by applying a load in the middle. The test can be applied to various materials, such as metals, plastics and composites with different section geometries. In the test, the material is considered as a simple beam model, and the aim is to have negligible shear stresses under ideal moment distribution [25]. The three-point bending test is a method of measuring the deformation that occurs when a circular or rectangular cross-section test piece rests freely on two supports and a force is applied to its center without changing direction (Figure 12).

In this study, the mechanical performances of different composite material types (GFRP and CFRP) were comprehensively investigated by three-point bending test using the same adhesive and joint geometry. The test process allows the determination of damage mechanisms occurring at the adhesive interface and joint area; in addition, the time-dependent development of changes in the mechanical properties of composite materials under the effect of seawater exposure is analyzed. The aim here is to systematically evaluate the damage formation and progression processes of bonded GFRP and CFRP single-lap composite joints aged in seawater environment by three-point bending test.

In a 3-point bending test on a simple beam, the maximum stress occurs at the midpoint where the load is applied. The bending moment is at its maximum at that point. The stress increases with both the bending moment and the distance from the neutral axis. Therefore, the highest stress occurs in the outermost fibers of the beam, at the midpoint. This represents the area where the material is most likely to fail under bending.

The following equation is used to calculate the stress developed at any point on the load-deflection curve:

*Flexural Stress* (*σf*):

(1) $$\sigma_{f}=\frac{3PL}{2bd^{2}}$$

Symbols in the formula:

$\sigma_{f}$: stress in the outer fibers at midpoint, MPa

*P*: load at a given point on the load-deflection curve, N

*L*: support span, mm

*b*: width of beam tested, mm

*d*: depth of beam tested, mm

*Flexural Strain*, (*ε_f_*):

(2) $$\varepsilon_{f}=\frac{6Dd}{L^{2}}$$

Symbols in the formula:

$\varepsilon_{f}$: strain in the outer surface, mm/mm

*D*: maximum deflection at mid-span, mm

*L*: support span, mm

*d*: depth, mm

**Figure 13:**
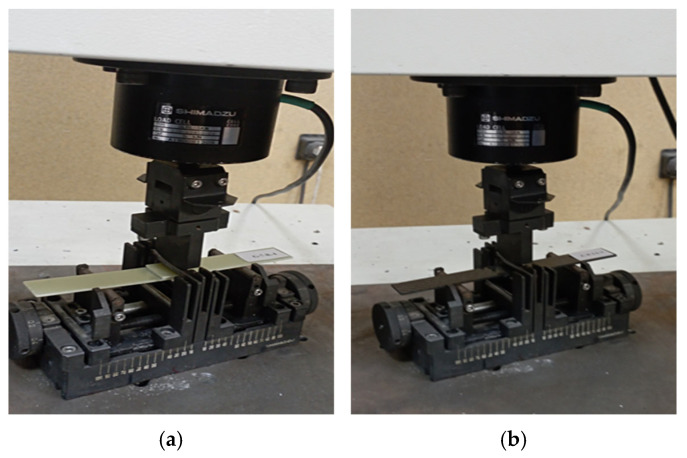
GFRP (**a**) and CFRP (**b**) specimen placed in the three-point bending test machine.

Three-point bending tests were performed at the Biomechanics Laboratory of the Department of Mechanical Engineering, Ege University, using a bending apparatus suitable for the Shimadzu AG-100 device (Shimadzu, Kyoto, Japan), with a capacity of 100 kN, at a load of 5 kN and a speed of 1 mm/min. The tests were performed on smooth GFRP and CFRP single-lap connections with an adhesive thickness of 0.2 mm according to the ASTM D790 standard [26]. The load, speed and sample dimensions are predefined in the test device, and the necessary calculations are performed automatically. The samples were tested both in seawater environment and in dry conditions, and the stress–strain curves were obtained by the relevant software. During the three-point bending test, the tensile stress occurs in the convex region, the compressive stress in the concave region, and the shear stress in the central axis of the sample. In order to minimize these stresses and ensure that the samples are kept in a balanced and symmetrical position on the test device, the gap between the beds is adjusted precisely and the samples are centered (Figure 13).

The experiment included a total of 36 connection samples, 6 of which were dry (not stored in sea water) and 15 GFRP and 15 CFRP samples stored in sea water.

## 3. Results

Before weight measurement, free moisture on the surfaces of GFRP and CFRP single lap jointed specimens was carefully removed using moisture absorbing paper to ensure measurement accuracy. Then, according to the data presented in Table 5 and Table 6, the moisture retention rates of the specimens were calculated using Equation (3) below.

M(%) = (m_y_ − m_k_)/(m_k_) × (100) (3)

In the equation, m_k_ represents the dry weight (g) of the sample measured before exposure to any moisture; my represents the wet weight (g) measured after being kept in seawater for a certain period of time. M represents the moisture retention rate of the sample in percentage. The moisture retention rates of GFRP and CFRP single lap joint specimens were evaluated by comparing them with reference samples stored in a dry environment (Table 5 and Table 6). All specimens were immersed in seawater at 23.5 °C for durations of 1 month (720 h), 2 months (1440 h), 3 months (2160 h), 6 months (4320 h) and 15 months (10,800 h). For the GFRP samples, the average moisture retention rates at these intervals were determined as 0.66%, 3.43%, 4.16%, 4.65% and 4.83%, respectively. In the CFRP samples, the corresponding values were found to be 0.57%, 0.86%, 0.87%, 0.92% and 0.96%. These results indicate that moisture absorption increases with longer seawater exposure in both materials, with GFRP exhibiting a higher moisture uptake compared to CFRP.

**Figure 14:**
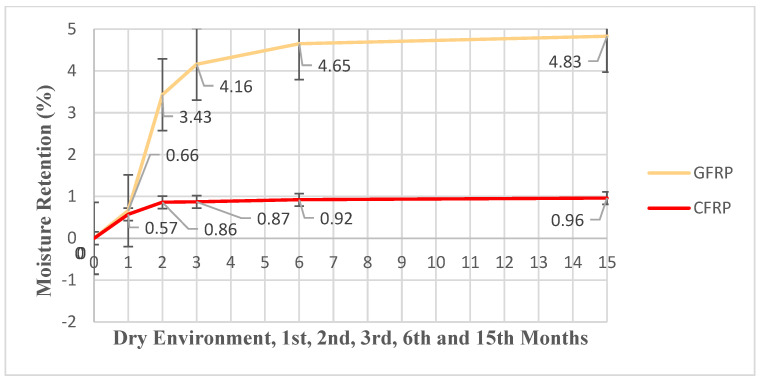
Moisture retention rates of GFRP and CFRP single lap joints in dry environment and sea water for 1, 2, 3, 6 and 15 Months (Between 720–10,800 h).

The comparative moisture retention rate graph of GFRP and CFRP samples is given in Figure 14.

Figure 14 presents the changes in moisture retention rates of GFRP and CFRP single lap joint samples depending on the duration of exposure to seawater.

According to Figure 14:

*Month 1 (720 h):* At the end of month 1, the average moisture retention rate in GFRP samples was measured as 0.66%. In this period, the rate was determined as 0.57% in CFRP samples. Both materials initially absorbed low levels of moisture, but it was observed that CFRP tended to absorb less moisture.

*Month 2 (1440 h):* In month 2, the moisture retention rate in GFRP samples increased to 3.43%. This significant increase shows that GFRP is more sensitive to environmental moisture. In the same period, CFRP samples retained only 0.86% moisture. This reveals that CFRP is more resistant to moisture effects.

*Month 3 (2160 h):* As of month 3, the moisture retention rate in GFRP samples reached 4.16%, while this value remained almost constant at 0.87% in CFRP. At this point, it was observed that CFRP was approaching the saturation level, and GFRP continued to absorb water.

*Month 6 (4320 h):* In month 6, GFRP samples retained 4.65% moisture, while this rate was 0.92% in CFRP. The increase in GFRP continues, although it has slowed down. It is seen that CFRP continues to absorb moisture at a very low rate.

*Month 15 (10,800 h):* At the end month 15, the moisture rate in GFRP samples reached its highest level, at 4.83%. CFRP, on the other hand, almost fixed its moisture intake at 0.96%. These results clearly show that CFRP is more stable and durable against moisture in the long term.

An increase in moisture absorption was observed in both materials over time; however, this increase was much more pronounced in GFRP samples. The rapid increase in moisture in GFRP, especially in the first two months, indicates the sensitivity of the material to sea water, while the slow and limited increase in CFRP shows that this material is highly resistant to water. These data show that GFRP samples absorb more moisture over time, while the moisture retention rates of CFRP samples remain quite low and limited.

### 3.1. Moisture Diffusion in Composite Materials

Moisture diffusion in composite materials plays a critical role, especially in terms of structural strength and long-term performance. Fick’s diffusion theory provides a basic approach to understanding this process. Fick’s laws allow for quantitative modeling of molecular diffusion, and in this context, the effect of the internal structural properties of composite structures on moisture transport can be evaluated in more detail [27].

Fick’s first law states that, under the assumption of constant temperature and constant diffusion coefficient, the flux density (D) in a system is proportional to the concentration gradient. However, in practical applications, especially in polymer matrix composites, the diffusion coefficient (D) may not be constant. This situation arises due to the effects of environmental conditions, such as morphological heterogeneities within the material, temperature changes and relative humidity. Therefore, time and location-dependent changes in the diffusion coefficient or concentration gradient must be taken into account [28].

Fick’s second law describes how the diffusion process evolves with time and is used to model concentration distributions that vary with time. The second law allows more accurate and realistic results to be obtained, especially in systems with time-varying or position-dependent diffusion coefficients. In composite materials, microstructural elements such as matrix-fiber interface, voids and cracks can significantly affect the diffusion behavior of moisture. Therefore, the use of extended or modified forms of Fickian diffusion theory contributes to a more accurate modeling of moisture transport in such materials [29,30]. Fick’s second law of diffusion is used to describe the time-dependent behavior of the diffusion process by taking into account the time-varying concentration profiles. It represents the application of Fick’s second law of diffusion to moisture diffusion in composite materials. This law is fundamental to model the evolution of moisture or liquid absorption over time, especially when composite materials are exposed to environmental conditions (e.g., immersion in water) [27].

- *Calculation of Diffusion Coefficient (D):*

(4) $$D=\pi {\left(\frac{h}{4M_{\infty }}\right)}^{2}{\left(\frac{M_{2}-M_{1}}{{\sqrt{t}}_{2}-\sqrt{t_{1}}}\right)}^{2}{\left(1+\frac{h}{L}+\frac{h}{w}\right)}^{-2}$$

This equation is used to calculate the diffusion coefficient (*D*) as a result of immersing a composite sample in water.

In the equation:

*D*: Diffusion coefficient (mm^2^/s),

*h*: Thickness of the sample (m),

*L*: Length of the sample (m),

*w*: Width of the sample (m),

*M_1_* and *M*_2_: Moisture contents measured at times *t*_1_ and *t*_2_ (kg),

*M∞*: Maximum mass change reached at the end of infinite time (kg),

*t*_1_ and *t*_2_: Time (s).

In composite materials with Fickian diffusion behavior, the diffusion coefficient can be calculated by taking into account the geometric dimensions of the sample and the moisture contents measured at two specific time intervals [9].

- *Theoretical Mass Change (M):*

(5) $$M=\left[1-\frac{8}{\pi^{2}}\exp\left(-\pi^{2}\frac{D_{t}}{h^{2}}\right)\right]M\infty$$

Equation (5) is based on the solution of Fick’s second law and is used to model how moisture uptake progresses with time. In particular, in the early stages (at small *t* values), uptake is rapid, slowing down with time and approaching *M*∞ asymptotically [27].

Using these two equations together, it is possible to calculate:

- The rate of moisture diffusion in composite materials (*D*),
- The moisture uptake behavior over time (*M*),
- The effect of material geometry on this process.

The mass increase and the change in moisture uptake form a linear relationship with time, gradually slowing down at a certain point. This process is usually associated with Fick’s law of diffusion. In this context, moisture uptake occurs when water molecules inside the composite material diffuse from the external environment into the material [31]. However, this process becomes more complex over time. Initially, the material can rapidly absorb water, but the accumulation of salt compounds on the surface slows down the rate of absorption. Salt deposits can cover the surface of the material, inhibiting or limiting the diffusion of water. Consequently, moisture uptake is often modeled by the square root function, which tends to slow down over time. This shows an initial rapid increase in moisture uptake, followed by a gradual decrease until a certain equilibrium point or saturation is reached [32,33].

This is especially important for materials in contact with seawater. Salts contained in seawater can accumulate on the surface of the material, preventing moisture absorption and changing the mechanical properties of the material. This effect leads to an increase in mass over time, and as the density of the salts increases, the water in the material becomes immobile [34,35]. In Figure 14, GFRP samples, the moisture retention rate increased from approximately 0.66% to 4.83% from 1 month to 15 months. In contrast, in CFRP samples, the moisture retention rate increased from only 0.57% to 0.96% during the same period. This shows that GFRP exhibits a more permeable structure to seawater and absorbs more moisture compared to CFRP.

First, in order to understand the Fickian behavior, the initial stages of moisture absorption were considered. In the time-dependent mass change curve seen in Figure 14, it was observed that moisture absorption exhibited Fickian diffusion behavior only in the initial stages. This means that the material initially rapidly absorbs seawater, and the mass increase increases proportionally with time. Before the saturation point was reached, the moisture-induced mass increase observed in the samples showed a linear increasing trend after a certain period of time. It was revealed that both GFRP and CFRP connection specimens showed a non-Fickian moisture absorption pattern. This non-Fickian behavior can be caused by salt deposits, matrix swelling and changes in the internal structure of the material. Such factors slow down the absorption of water. In Figure 14, the mass increase in the GFRP connection continues for a longer time compared to the CFRP connection. This difference is due to the differences in the internal structure of the materials. The more flexible and porous structure of GFRP allows water to diffuse more easily, and the swelling of the matrix becomes more pronounced. This swelling allows more moisture to move within the material and allows diffusion to continue.

On the other hand, the harder and lower porosity structure of CFRP causes moisture to move less within the material [36,37]. This difference causes the moisture uptake of GFRP to continue for a longer time and CFRP to reach saturation at an early stage.

In this study, the immersion temperature was determined only as room temperature. This makes the calculations of the diffusion coefficient (D) particularly difficult because the diffusion coefficient changes proportionally with temperature. At room temperature, the mobility of molecules will be lower compared to higher temperatures, and the rate at which the material absorbs moisture will be limited. Therefore, the diffusion coefficient at room temperature is often considered a characteristic parameter corresponding to the permeability index of the material. The permeability index is the resistance of the material to the passage of water vapor or liquid, and can be used as a parameter to evaluate the overall performance of the material [38].

As is known, the low increase in the moisture retention rate of CFRP is due to its denser fiber structure, the resin system’s lower water permeability, and the increase resistance to water caused by the chemical structure of carbon fibers [39,40]. These results showed that CFRP single lap connected specimens are a more suitable material in terms of long-term strength in seawater.

### 3.2. Three Point Bending Test Results

- *Dry Environment Samples (G-7-K and C-8-K)*

**Figure 15:**
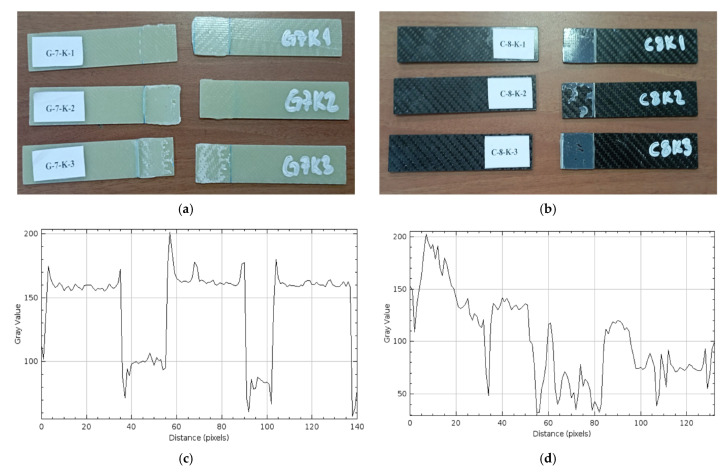
G-7-K Dry Environment (**a**), C-8-K Dry Environment (**b**), Roughness Profile of Bonded Surfaces GFRP (**c**) and CFRP (**d**).

Figure 15 presents three samples demonstrating the damages observed after the three-point bending test of GFRP samples kept in a dry environment as well as the CFRP samples. Samples stored in dry conditions were used as references in the comparative evaluation with samples exposed to sea water. These reference samples were stored at 23.5 °C without contact with sea water. Therefore, the damages observed in these samples occurred due to the mechanical effects of the three-point bending test.

In Figure 15a, it is observed that the G-7-K-1 and G-7-K-2 samples are separated from each other starting from the bonding bond line. Such separations are due to the stress accumulation concentrated at the ends of the sample due to the bending stress applied in the three-point bending test. This separation was seen more clearly in the sample labeled as G-7-K-3, especially compared to the G-7-K-1 and G-7-K-2 samples. The general surface structures of all the samples in Figure 15 maintained their smoothness, and there was no displacement of the fibers on the laminate structure or visible separation in the outer layers. After the bending load, the damage areas were concentrated at the ends, and the integrity was preserved throughout the body. This situation reveals that the highest stress during loading was collected at the lower surface end points, and the fracture behavior started at these points. As a result, it was seen that the damages observed in the GFRP samples kept in a dry environment were only due to the three-point bending test.

In Figure 15b, ruptures were detected starting from the adhesive bond line in C-8-K-2 and C-8-K-3 samples. These ruptures occurred as a result of the load applied in the bending test. The surface structures of the CFRP samples largely preserved their brightness and smoothness. This can be associated with being a dry sample and the structural stability of the CFRP material.

As a result of the profile analysis performed on GFRP single-lap bonding in Figure 15c, sudden and high-amplitude gray value changes were observed on the surface. This shows that the surface roughness is high and there is irregularity in the bonding areas. On the other hand, lower-amplitude and more regular gray value changes were observed on the CFRP surface profile in Figure 15d, which revealed that the surface was more homogeneous and smooth.

- *Samples Soaked in Sea Water for 1 Month (720 h) (G-7-1A and C-8-1A)*

**Figure 16:**
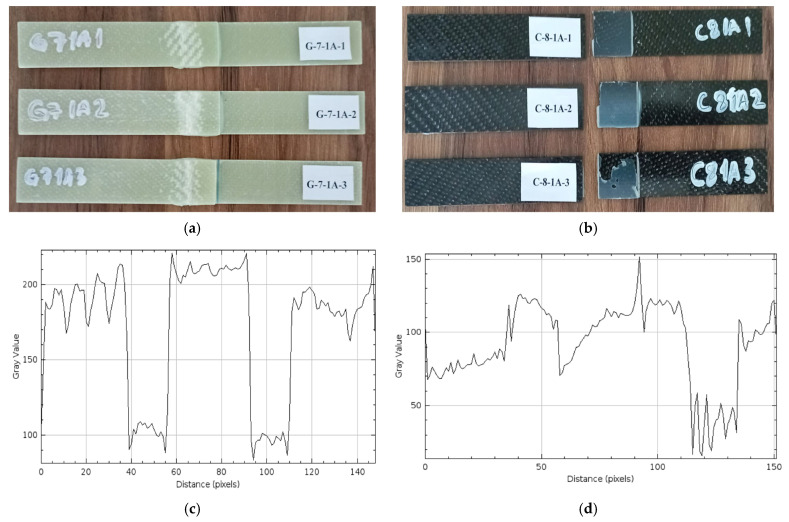
G-7-1A 1 month (720 h) (**a**), C-8-1A 1 month (720 h) (**b**), Roughness profile of bonded surfaces GFRP (**c**) and CFRP (**d**).

**Figure 17:**
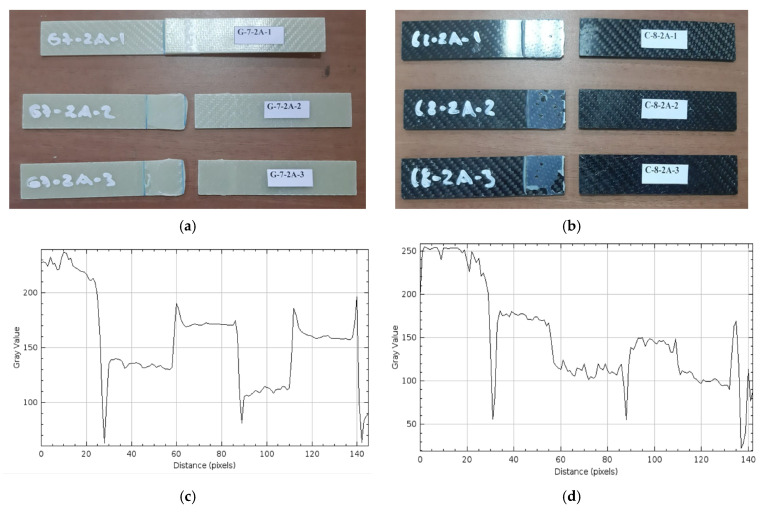
G-7-2A 2 months (1440 h) (**a**), C-8-2A 2 months (1440 h) (**b**), Roughness profile of bonded surfaces GFRP (**c**) and CFRP (**d**).

Regarding the damages observed after the three-point bending test, three different samples of GFRP samples held for 1 month (720 h) are presented in Figure 16, and Figure 17 presents the same condition on CFRP samples.

In Figure 16a, samples kept in sea water for 1 month, no rupture occurred starting from the bonding line. The whitish areas in the middle parts of the samples are remarkable. These separations can be explained by the ductility of GFRP under the applied mechanical load.

In Figure 16b, C-8-1A-1 and C-8-1A-2 show damage in the rupture at the adhesive bond line. The rupture observed in the middle region of the C-8-1A-1 and C-8-1A-2 samples shows that the damage was sudden. In the C-8-1A-3 samples, the adhesive layer remained on the surface of the sample. The sudden rupture is due to the high structural strength of the carbon fibers and the superior resistance of the joint to seawater. However, local ruptures are observed at the ends of the CFRP samples. These ruptures developed due to mechanical loading.

In the profile analysis performed after 1 month of seawater storage, high amplitude and sudden fluctuations were observed in the gray values of the GFRP single-lap bonding surface in Figure 16c. This situation indicates deterioration due to swelling, cracks or adhesion loss caused by water on the surface. On the other hand, in Figure 16d, a lower level of roughness was generally observed on the CFRP surface, but a significant surface deterioration was detected in the range of 90–130 pixels. These results show that the CFRP connection is more resistant to seawater.

- *Samples Soaked in Sea Water for 2 Months (1440 h) (G-7-2A and C-8-2A)*

The damages observed after the three-point bending test on three different GFRP samples held for 2 months (1440 h) are presented in Figure 17a, and on CFRP samples in Figure 17b.

In Figure 17a, it is observed that the G-7-2A-1 sample did not break. In the G-7-2A-2 sample, the fracture surface in the connection is more pronounced; the adhesive traces on one side of the interface are particularly noticeable. The G-7-2A-3 sample broke in the connection and exhibited deformation.

In Figure 17b, it was observed that in the C-8-2A-1 sample, although the surface remained generally intact, there was a break in the bonding area. In the C-8-2A-2 sample, it was observed that the layer was separated from the bonding surface in places, and bonding gaps formed on the surface and in the sample. In the C-8-2A-3 sample, obvious adhesive traces were observed in the bonding area.

In Figure 17c, the gray value profile of the GFRP sample shows an irregular and wavy structure. Gray values vary between 250 and 50 and decrease intensely, especially after the 20th pixel, exhibiting ups and downs at many points. These high-frequency fluctuations in the graph show that the homogeneity on the GFRP surface is disrupted, the fiber–matrix interaction is weakened, and seawater penetration is effective. In addition, the sudden transitions seen in the middle and end parts of the graph show that the surface is cracked or swollen at a microscopic scale. In Figure 17d, the gray value profile of the CFRP surface exposed to seawater for 2 months has a generally controlled structure, although it shows irregularity in certain areas. The gray values vary between approximately 250 and 100. The decreases observed in the graph indicate surface defects, such as interface separation, microcracks caused by salt effect or resin deformations. However, the stable areas following the decreases reveal that the surface still maintains its homogeneity in some areas, and that the CFRP exhibits this resistance.

- *Samples soaked in sea water for 3 months (2160 h) (G-7-3A and C-8-3A)*

**Figure 18:**
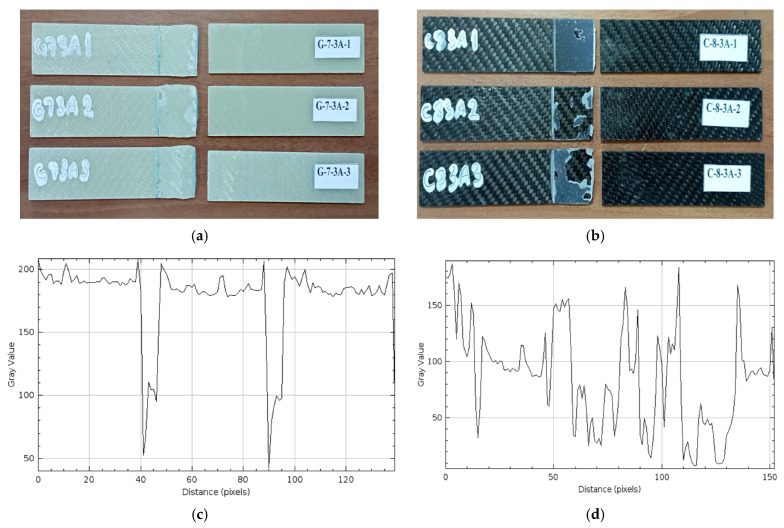
G-7-3A 3 months (2160 h) (**a**), G-7-3A 3 months (2160 h) (**b**), Roughness profile of bonded surfaces GFRP (**c**) and CFRP (**d**).

The damages observed after the three-point bending test in three different GFRP samples held for 3 months (2160 h) are presented in Figure 18a, and on CFRP samples in Figure 18b.

In Figure 18a, it was observed that the damage in sample G-7-3A-1 occurred in the form of a rupture directly at the bonding line. In sample G-7-3A-2, it was determined that the damage remained on the surfaces in the form of bonding failure. In sample G-7-3A-3, a mixed damage type occurred, with both rupture at the bonding line and adhesive residues on both surfaces. In the first two samples (G-7-3A-1 and G-7-3A-2), it was observed that the damage was concentrated on the upper surface, while in sample G-7-3A-3, it was determined that the damage spread to both surfaces.

In Figure 18b, it is observed that, in the C-8-3A-1 sample, the breakage occurred in the majority of the adhesive layer in the connection area, but no serious damage was observed. In the C-8-3A-2 sample, the most separation symptoms were seen in the connection samples. In the C-8-3A-3 sample, the remaining adhesion effect was seen.

In Figure 18c, although a generally horizontal and stable line is observed in the gray level profile of the GFRP sample, there are noticeable decreases in some areas. Gray values vary between 200 and 50. There are very sharp decreases especially around the 40th, 85th and 100th pixels. These sudden decreases indicate defects such as local delaminations, salt crystal accumulation and micro cracks on the surface. In Figure 18d, the gray level graph of the CFRP sample is quite irregular. Gray values fluctuate between 150 and 20, and many sudden increases and decreases are observed along the profile. This indicates that microscopic irregularities and deteriorations occur in the adhesive bond on the surface.

Although there is an increase in the adhesive surface roughness at certain points in the CFRP exposed to sea water for 3 months, the results show that CFRP can maintain its strength even if it experiences partial surface deterioration over time in the sea environment.

- *Samples Soaked in Sea Water for 6 months (4320 h) (G-7-6A and C-8-6A)*

**Figure 19:**
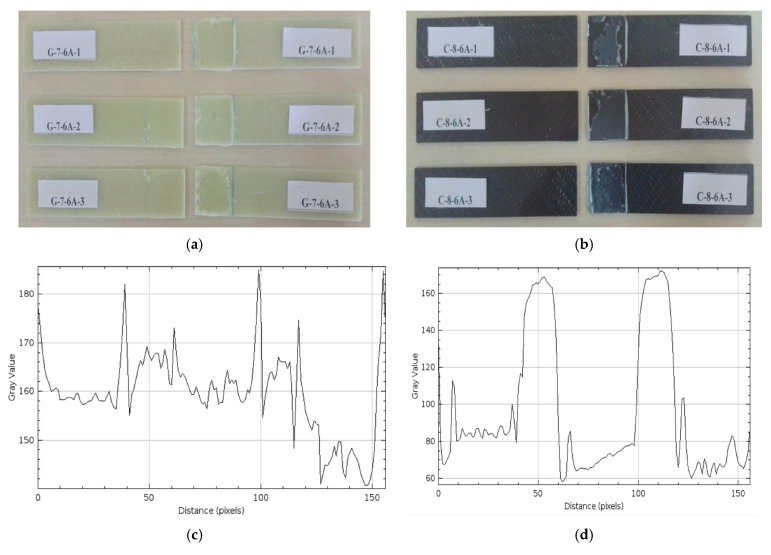
G-7-6A 6 months (4320 h) (**a**), C-8-6A 6 months (4320 h) (**b**), Roughness Profile of Bonded Surfaces GFRP (**c**) and CFRP (**d**).

The damages observed after the three-point bending test on three different GFRP samples held for 6 months (4320 h) are presented in Figure 19a, and on CFRP samples in Figure 19b.

In Figure 19a, it is seen that there are breaks along the bond line in the connection areas of samples G-7-6A-1 and G-7-6A-2. In contrast, more pronounced dispersions are observed in the bond line in the connection area in sample G-7-6A-3. This situation shows that since the glass fiber reinforced structure is relatively more permeable to sea water, moisture diffuses into the resin and negatively affects the bond quality.

In Figure 19b, adhesive cracks were detected on the bonding surfaces of the C-8-6A-1 and C-8-6A-2 connection samples. In the C-8-6A-3 sample, it was observed that the bonding layer broke off and separated in the bonding area. These situations show that, although the carbon fiber reinforced structure is more rigid, the connection bonding areas are weakened by the effect of sea water.

The gray level graph of the GFRP sample in Figure 19c shows a much more irregular profile compared to the CFRP. This irregularity indicates the intense deterioration and layer separation, as well as the apparent delamination in the bonding areas and weakening of the epoxy binder that occurred over time on the GFRP surface. These deteriorations on the surface show that the bonding quality has decreased significantly. On the other hand, the gray level graph of the CFRP sample shown in Figure 19d indicates that the values vary between 150 and 185, and generally reveals a more regular and controlled surface profile. The distinct sharp peaks seen in the graph represent local adhesive residues and roughness on the surface. This situation shows that there is limited deterioration on the CFRP surface, but the overall bonding quality is maintained.

- *Samples Soaked in Sea Water for 15 months (10,800 h) (G-7-15A and C-8-15A)*

**Figure 20:**
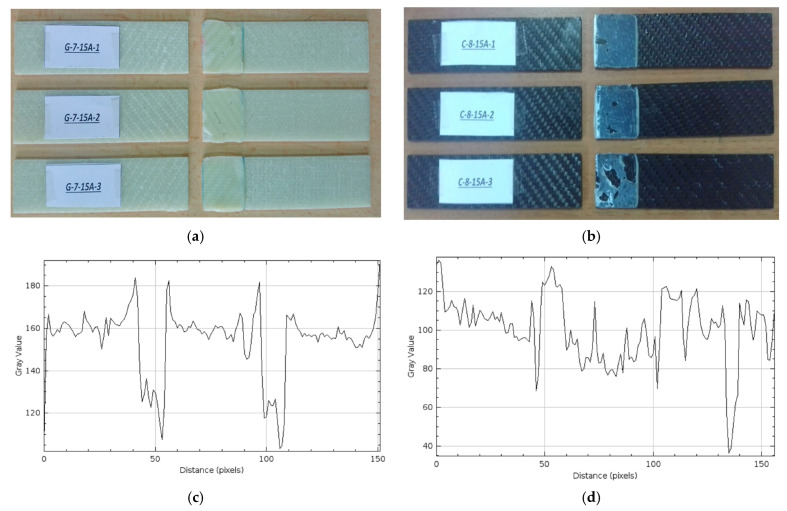
G-7-15A 15 months (10,800 h) (**a**), C-7-15A 15 months (10,800 h) (**b**), Roughness profile of bonded surfaces GFRP (**c**) and CFRP (**d**).

The damages observed after the three-point bending test in three different GFRP samples held for 15 months (10,800 h) are presented in Figure 20a, and on CFRP samples in Figure 20b.

In Figure 20a, adhesion failures were detected in the connection areas of samples G-7-15A-1, G-7-15A-2 and G-7-15A-3. However, white spots were observed in sample G-7-15A-1, which is an indication of damage caused by mechanical loading.

In Figure 20b, in the C-8-15A-1 and C-8-15A-2 samples, the samples broke off by remaining superficially attached at the bond line. In the C-8-15A-3 sample, the adhesive partially remained on the bonding surfaces, and brittle fracture was observed. The gray level graph of the GFRP sample kept in sea water for 15 months in Figure 20c generally has values varying between 120 and 185. The sudden drops and increases that are noticeable on the graph indicate that there is intense roughness, layer separation and epoxy deterioration on the bonding surface. The sharp fluctuations, especially in the 40–60 and 90–110 pixel ranges, can be associated with local delaminations and adhesion losses. The less resistant structure of GFRP to water caused significant physical deterioration in the bonding area during this period. The gray level graph of the CFRP sample in Figure 20d varies in a narrower range (approximately 40 to 130). Although the graph presents a more regular profile compared to GFRP, there are still significant ups and downs. The sudden drops, especially around 50 pixels and 130 pixels, indicate local surface deterioration and detachment of adhesive residues. However, the general profile reveals that CFRP shows less deterioration in the bonding zone compared to GFRP and is more resistant to seawater.

It was observed that GFRP samples showed lower resistance to the seawater effect compared to CFRP; the damage in the adhesive connection were more pronounced due to seawater absorption. This damage shows the diffusion effect of seawater causes the dissolved ions to diffuse into the bonding area and negatively affect the bond strength. Table 7 presents the damage conditions observed according to the seawater exposure time.

**Figure 21:**
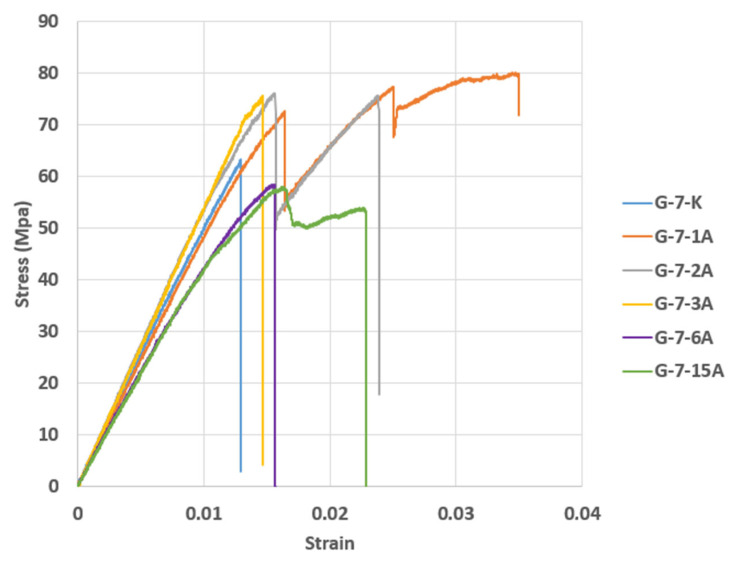
Stress–strain graphs of GFRP samples kept in seawater conditions for different periods (1, 2, 3, 6 and 15 months) and stored in dry conditions.

The stress–strain curves presented in Figure 21 were obtained as a result of bending tests performed after storing GFRP composite samples in a dry environment and in sea water for periods of 1, 2, 3, 6 and 15 months.

In Figure 21, the bending stress and strain values for specimen G-7-K were measured as 63.0726 MPa and 0.0129. In specimen G-7-1A, the initial bending stress was 72.2704 MPa and 0.0163. Following this, the lowest bending stress and strain value were 54.4886 MPa and 0.0113, and the highest values were measured as 80.0818 MPa stress and 0.0347 strain. In specimen G-7-2A, the initial and highest bending stress was 75.8136 MPa and 0.0156. Then, the stress decreased to 50.8163 MPa with a strain of 0.0094, and then increased to 75.6766 MPa at a strain of 0.0154. In the G-7-3A sample, the initial highest bending stress was measured as 75.6723 MPa, and the strain value was 0.0146. In the G-7-6A sample, the bending stress was measured as 58.4044 MPa, and the strain value was 0.0160. In the G-7-15A sample, the initial bending stress was 57.55439 MPa, and the strain value was 0.0159. Following this, the lowest bending stress was measured as 50.4088 MPa, the strain value was 0.0129, and the final value was measured as 53.4619 MPa stress and 0.0140 strain.

**Figure 22:**
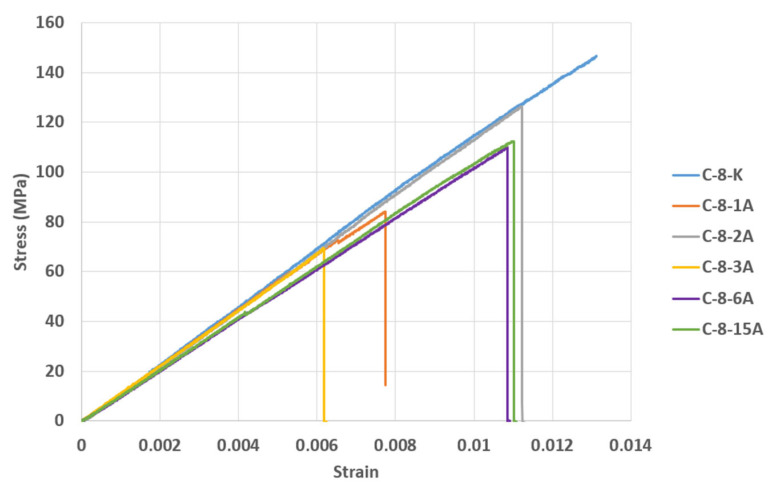
Stress–strain graphs of CFRP samples kept in seawater conditions for different periods of time (1, 2, 3, 6 and 15 months) and stored in dry conditions.

In Figure 22, the bending stress for the C-8-K sample was measured as 146.5976 MPa, and the strain value as 0.0131. In the C-8-1A sample, the initial bending stress was 72.4395 MPa, and the strain value was 0.0065. Following this, the highest bending stress was 83.6555 MPa, and the strain value was 0.0077. In the C-8-2A sample, the initial and highest bending stress was 126.2889 MPa, and the strain value was 0.0112. In the C-8-3A sample, the bending stress was 69.1586MPa, and the strain value was 0.0061. In the C-8-6A sample, the bending stress was 109.3453 MPa, and the strain value was 0.0107. In the C-8-15A sample, the initial bending stress was measured as 112.2266 MPa, and the strain value was measured as 0.0110.

Young’s Modulus, also called Modulus of Elasticity, is a fundamental mechanical property that measures the hardness or rigidity of a material. A high Young’s Modulus indicates that a material is very stiff, meaning it will deform less under a given load. Conversely, a low Modulus of Elasticity indicates that the material is more flexible [41].

The modulus of elasticity (Young modulus) was determined by calculating the slope of the linear region of the stress–strain curves in Figure 21, and the results are presented in Table 8. Similarly, the data in Table 9 were obtained using the slope of the linear part of the stress–strain curves in Figure 22. The obtained modulus of elasticity (E) values are detailed below.

### 3.3. Damage Analysis of GFRP and CFRP Composites Subjected to Bending Tests with SEM

**Figure 23:**
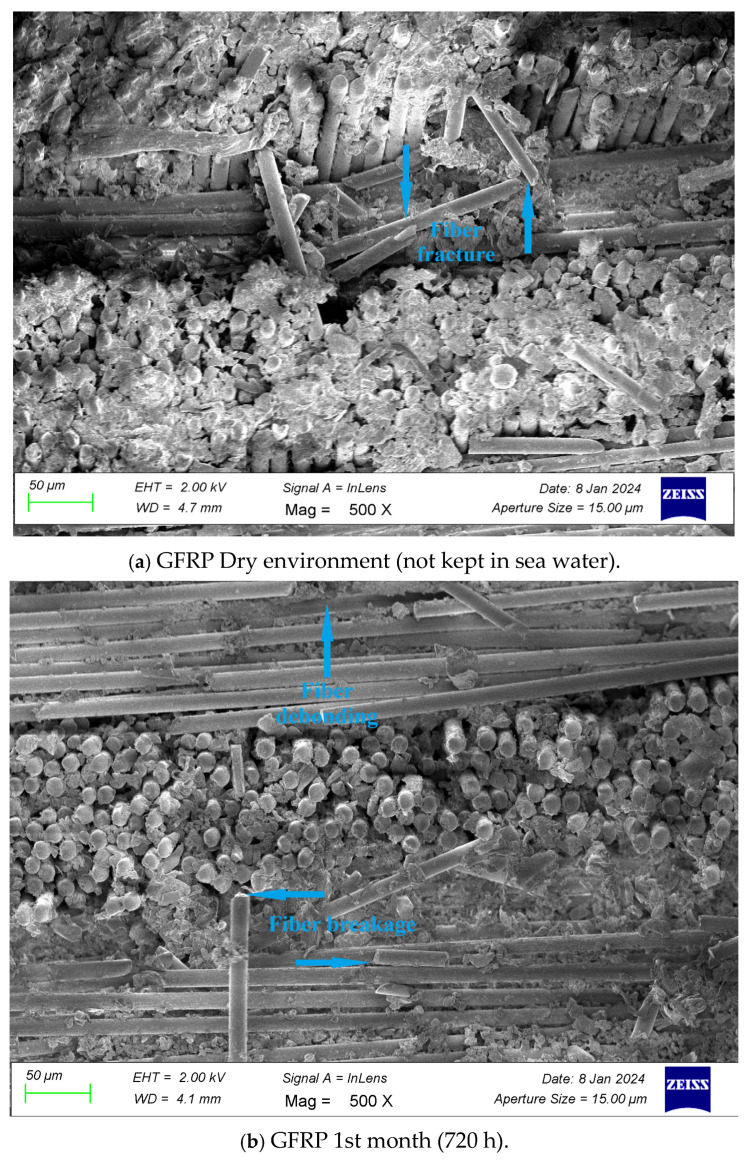

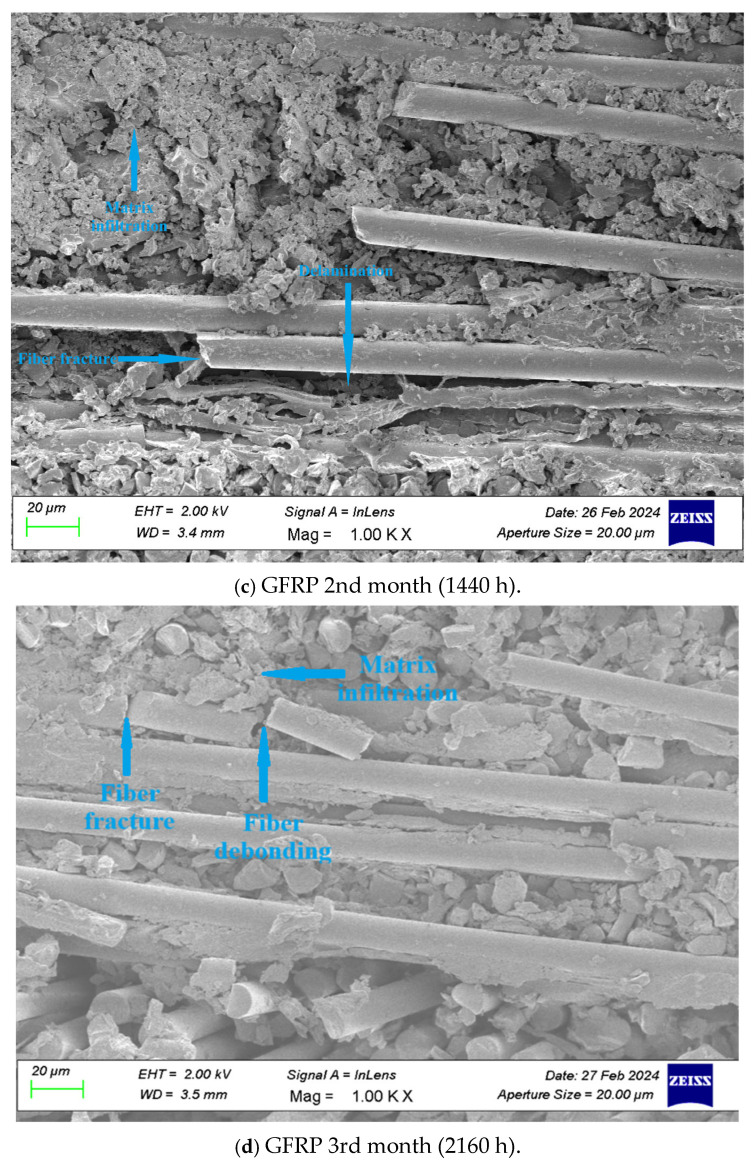

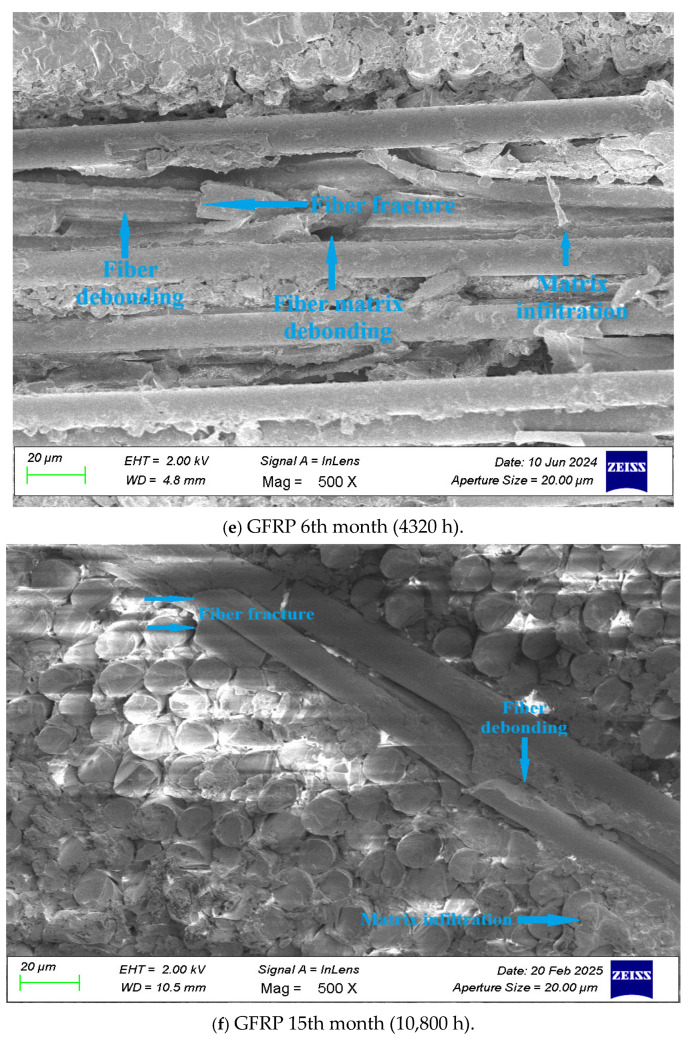
SEM image of GFRP composite material after three-point bending test.

As the storage period in seawater increases, the types of interlayer and joint damages should become more apparent. The damage may be due to various factors, such as material structure, joint type and adhesive used. Therefore, it is of great importance to analyze composite materials. Figure 23 show the SEM images (ZEISS GEMINI SEM 560, Dokuz Eylül University Center for Science and Technology Application and Research, İzmir, Türkiye) of GFRP specimens, respectively, after being subjected to three-point bending tests following exposure to seawater for 1 month (720 h), 2 months (1440 h), 3 months (2160 h), 6 months (4320 h), and 15 months (10,800 h), as well as in dry conditions.

***In GFRP SEM images;***

***In this case, in a dry environment (not kept in sea water),*** fiber fracture and fiber rupture damages were observed in GFRP samples as a result of mechanical loading. Since it was a dry environment, the fiber–matrix interface remained intact.

***In samples kept for 1 month (720 h),*** weakening of the fiber–matrix bonds, micro-deterioration on the matrix surface and slight fiber rupture and breakage were caused.

***In the samples held for 2 months (1440 h),*** fiber breakage, fracture, matrix deterioration and delamination (delamination) were observed. The fiber–matrix interface weakened, microstructure dispersion and surface roughness increased.

***In the samples held for 3 months (2160 h),*** fiber breakage and fracture continued; more pronounced separations occurred at the fiber–matrix interface. Although water absorption slowed down, structural deterioration progressed. Delamination increased.

***In the samples held for 6 months (4320 h),*** the matrix began to deteriorate and largely lose its binding properties. The adhesion between the fibers weakened, and the fibers became easily separable. Cracks became apparent in the microstructure.

***In the samples held for 15 months (10,800 h),*** the overall integrity of the GFRP structure was significantly damaged. The fibers were largely free. Delamination reached an advanced level.

Table 10 presents the bending test data and SEM analysis obtained according to the different exposure times of GFRP connection samples in the dry environment and sea water.

**Figure 24:**
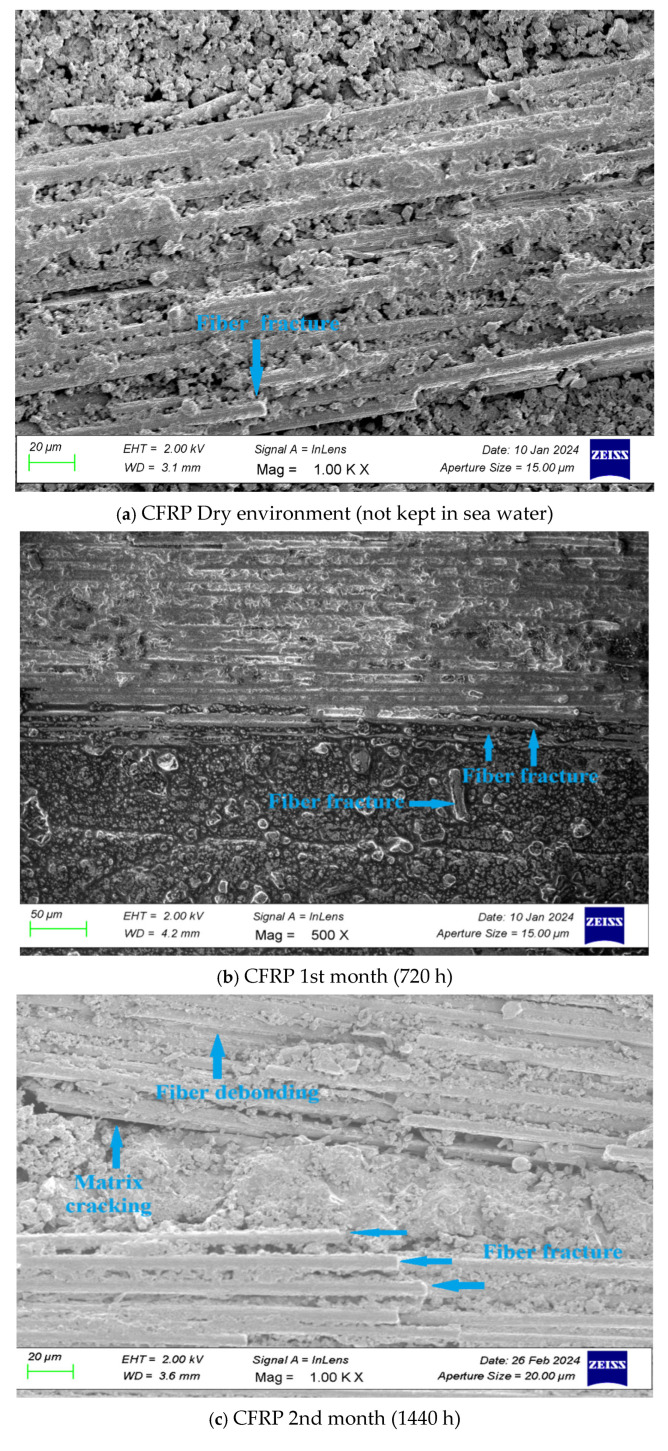

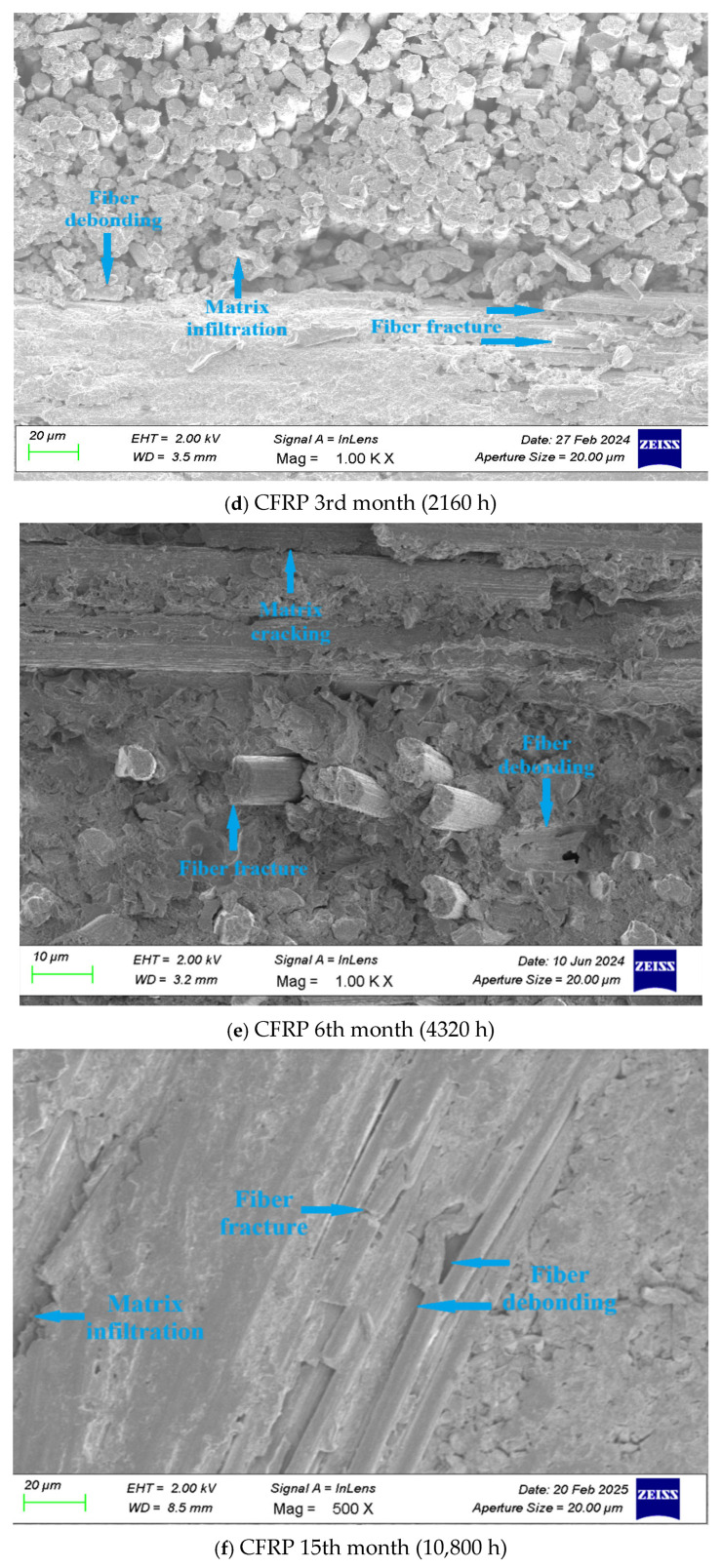
SEM image of CFRP composite material after three-point bending test.

Figure 24 show the SEM images (ZEISS GEMINI SEM 560, Dokuz Eylül University Center for Science and Technology Application and Research, İzmir, Türkiye) of CFRP specimens, respectively, after being subjected to three-point bending tests following exposure to seawater for 1 month (720 h), 2 months (1440 h), 3 months (2160 h), 6 months (4320 h), and 15 months (10,800 h), as well as in dry conditions.

***In CFRP SEM images;***

***In the structure of the CFRP sample that was not kept in a dry environment (sea water),*** the fibers were aligned properly, and they exhibited a strong bond with the polymer matrix. The matrix layer showed a homogeneous distribution, and no separation or voids were detected at the fiber–matrix interfaces. However, fiber breaks were observed due to the effect of mechanical loading.

***In the samples that were kept for 1 month (720 h),*** initial deteriorations in the microstructure due to seawater absorption were detected. Irregularities and slight surface abrasions were observed on the matrix surface. Small voids and separations also occurred around the fibers. Slight fiber breaks occurred.

***In the samples that were kept for 2 months (1440 h),*** various microstructural damages were detected in the material structure. Micro cracks and deteriorations were observed in the resin matrix, which occurred due to the penetration of moisture and salt in sea water into the matrix. Separations and fiber breaks occurred at the fiber–matrix interfaces.

***In the samples kept for 3 months (2160 h),*** significant cracks and local separations were observed in the matrix structure. Separation at the fiber–matrix interface increased, some carbon fibers began to surface. In addition, micropores were formed due to seawater absorption.

***In the samples kept for 6 months (4320 h),*** there were significant separations at the fiber–matrix interface; micropores accelerated, the structure absorbed more moisture, and fiber breakage was observed. The loss of structural integrity of the matrix and the dislocation of the fibers indicate that the mechanical strength began to decrease. In addition, irregularities on the fracture surface reveal that the brittle fracture behavior increased.

***In the samples kept for 15 months (10,800 h),*** it was determined that the microstructure of the structure deteriorated. Separation and surface wear were observed in the polymer matrix phase; the matrix, which lost its binding properties, significantly weakened the interface bonds with the carbon fibers. There was significant separation at the fiber–matrix interfaces, the structural integrity of the carbon fibers was disrupted, and fiber breaks occurred in some areas. In addition, pore formation and micro-voids are noticeable in the inner regions of the structure, which clearly shows the diffusion of seawater into the material and moisture absorption.

The comparison table of the bending test data and SEM analysis obtained according to the different exposure times of CFRP connection samples in dry environment and sea water is given in Table 11.

When the SEM analyses of the samples kept in sea water are examined, while superficial damages, matrix cracks and fiber separations are mostly observed in CFRP samples, deeper structural damages such as fiber–matrix bond rupture, microscopic delaminations and fiber breaks are prominent in GFRP samples. This result shows that, especially in GFRP samples, sea water diffuses into the structure and causes moisture absorption, weakens the bond strength, and decreases mechanical strength.

One of the main reasons for the performance loss observed in composite materials as a result of moisture intake and exposure to environmental factors is the changes that occur in the physical and chemical properties of the resin matrix. The diffusion of water molecules between polymer chains induces both physical and chemical changes in the matrix structure, leading to a reduction in the glass transition temperature (Tg) and a weakening of the intermolecular interactions. This condition may result in the degradation of the mechanical properties of the material. In this context, softening (plasticization) occurs in the matrix resin under the influence of moisture. As the mechanical hardness and strength of the matrix decrease, the load carrying capacity decreases [9].

The softening of the matrix adversely affects not only its own structural integrity but also the interfacial bonding strength between the fiber and the matrix. This deterioration in the fiber–matrix adhesion may lead to a reduction in the overall mechanical performance of the composite material. When moisture penetrates the interfacial region, micro-voids and microcracks can form in this region. This prevents effective load transfer at the points where the fibers should take on the load-carrying task [42]. The weakening of interfacial bonds reduces the matrix’s ability to effectively absorb shear stresses, thereby compromising the stress distribution mechanism between the fiber and the matrix within the composite structure. This causes the load to be concentrated in the matrix without being sufficiently transmitted to the fibers, leading to premature failure [43].

The results show that CFRP materials present more stable stress–strain curves and mechanical performance loss is more limited compared to GFRP, especially against long-term marine environment exposure. GFRP, on the other hand, was subjected to more deformations due to factors such as matrix swelling, plasticization and interface failures due to high water absorption, which resulted in a decrease in structural rigidity and load carrying capacity.

## 4. Discussion

Most of the existing studies on adhesively bonded single lap joints in composite materials have only provided evaluations within a single composite material type and limited environmental conditions. This has prevented a sufficient understanding of the long-term effects of aggressive environments, especially seawater, and the interactions between different composite material types. In the literature, the relationship between seawater-induced bending stresses and joint performance has been largely ignored. Studies have generally related the bending strength of composite joints only to material properties, and the contribution of seawater absorption to the microstructural damage process and joint behavior has been addressed to a limited extent. Therefore, holistic evaluation of the responses of different composite systems is of critical importance in terms of reliability and durability in applications.

In the study, composite joints bonded with single-lap adhesive were kept in separate containers in natural seawater taken from the Aegean Sea, which has a salinity of 3.3–3.7% and is kept at a temperature of 23.5 °C, for 1 month (720 h), 2 months (1440 h), 3 months (2160 h), 6 months (4320 h) and 15 months (10,800 h).

During these periods, the moisture retention percentages of the samples were measured at certain intervals, and then three-point bending tests were applied on each sample to evaluate their mechanical strength. The microstructural damages and degradations that occurred were thoroughly examined using Scanning Electron Microscopy (SEM). This method enabled a comprehensive evaluation of the effects of seawater exposure duration on the damage mechanisms and their influence on the structural performance of the composite joints.

Thus, seawater absorption on different composite materials (GFRP and CFRP) bonded with single-lap adhesive used in the marine environment was investigated. Specifically, this study attempted to determine when long-term exposure to seawater triggered microstructural damage, and when the effects of such damage on mechanical properties become apparent. Experimental results clearly demonstrate the differences in moisture retention rates between GFRP and CFRP single lap adhesive joints. While the initial 1 month (720 h) moisture retention rate of GFRP joints in dry conditions was 0.66%, this rate increased to 3.43% at 2 months (1440 h), 4.16% at 3 months (2160 h), 4.65% at 6 months (4320 h) and 4.83% at 15 months (10,800 h) of exposure to seawater, respectively. On the other hand, in CFRP samples, the moisture retention rate under dry conditions was measured as 0.57% at the beginning of 1 month (720 h); with seawater exposure, this rate reached 0.86% at 2 months (1440 h), 0.87% at 3 months (2160 h), 0.92% at 6 months (4320 h) and 0.96% at 15 months (10,800 h). These data clearly shows that GFRP material has a higher tendency for seawater absorption compared to CFRP. The rapid increase in the moisture retention rate of GFRP connection samples indicates that this material will be more affected by seawater in long-term use. On the other hand, CFRP connection samples showed that they can maintain their structural integrity better in the long term due to lower moisture absorption. According to the three-point bending test results, the Young modulus of the GFRP single lap joint specimens kept in seawater for 1 month (720 h), 2 months (1440 h), 3 months (2160 h), 6 months (4320 h) and 15 months (10,800 h) decreased by 5.94%, 8.90%, 12.98%, 24.11% and 25.23%, respectively, compared to the reference specimens tested in dry conditions. This finding shows that the swelling and plasticization effects caused by water absorption in the matrix phase as a result of exposure of the GFRP material to humid environments significantly reduced the rigidity and carrying capacity of the material. On the other hand, the Young modulus of the CFRP single lap joint specimens exhibited a decrease of 1.28%, 3.39%, 3.74%, 9.21% and 11.13%, respectively, in the specimens stored in seawater for the same periods of time compared to the reference specimens. This result reveals that the CFRP material maintains its structural stability as its lower water absorption capacity and higher mechanical strength allows it to be less affected by environmental effects. In general, it appears that, as the seawater immersion time increases, the negative effects on the mechanical properties of CFRP remain at much lower levels compared to GFRP, although a decrease in Young’s modulus was observed for both materials. The findings obtained from Scanning Electron Microscope (SEM) analyses and three-point bending tests show that, in both GFRP and CFRP single-lap adhesively bonded samples, damage under mechanical loading was first initiated in the polymer matrix phase and then transferred to the fiber phase. The strain developed during three-point bending loading was recorded in parallel with the time-dependent change of the applied stress, and the deformation behavior of the materials was evaluated. The lower the level of strain, the higher the rigidity and the more resistant the structure was to deformation. In addition, as the immersion time in seawater increased, both composite types were exposed to microstructural changes due to environmental effects. However, the strain values observed in CFRP single lap joints remained at lower levels compared to GFRP joints tested under the same conditions. This indicates that the polymer matrix phase of CFRP is more resistant to water absorption, and the fiber–matrix interfacial bonds remain more stable. Thus, CFRP joints exhibited superior mechanical properties in terms of structural performance in high humidity and salt conditions, such as the marine environment.

## 5. Conclusions

Seawater absorption in adhesive single lap joints significantly affects the long-term mechanical performance of composites. The data show that GFRP joints absorb more moisture over time than CFRP joints, which accelerates microstructural degradation and significantly reduces mechanical strength. In contrast, CFRP joints maintain structural integrity more effectively due to lower moisture absorption and higher resistance to environmental damage. Three-point bending test results showed that the Young modulus of GFRP decreased by 25% and CFRP by 11% after 15 months of seawater exposure. This indicates that CFRP is more suitable for applications requiring durability in marine environments. Scanning Electron Microscopy further confirmed that the damage initiation occurs in the matrix and progresses to the fiber, with GFRP showing earlier and more severe damage than CFRP. Strain measurements supported the mechanical test results, indicating higher rigidity and structural stability in CFRP over time.

In summary, CFRP exhibits significantly better performance than GFRP under long-term seawater exposure, making it a more reliable choice for marine applications involving adhesively bonded joints.

## Figures and Tables

**Table 1:** Production features for GFRP and CFRP samples.

Property	GFRP (Glass Fiber Reinforced Polymer)	CFRP (Carbon Fiber Reinforced Polymer)
Number of Layers	7 layers (due to volume and density ratio)	8 layers (due to volume and density ratio)
Orientation	0/90	0/90
Weave Type	Twill	Twill
Areal Density (g/m^2^)	390	245
Fiber Type	390 g glass fiber weft yarn	3K carbon fiber weft yarn
Prepreg Production	Drum-type prepreg machine	Drum-type prepreg machine
Resin System	F-RES 21 epoxy + F-Hard 22 hardener	F-RES 21 epoxy + F-Hard 22 hardener
Laminate Thickness	2 mm	2 mm
Manufacturer	Fibermak Engineering Company (Izmir, Türkiye)	Fibermak Engineering Company (Izmir, Türkiye)

**Table 2:** Mixture properties.

Property	Specification
Mixing Ratio (by weight)	RES 21 Resin: 100 F-Hard 22 Hardener: 21
Mixture Viscosity (at 25 °C)	500–800 mPa·s
Prepreg Curing	120 °C for 60 min

**Table 3:** Post-cured properties.

Property	Value (MPa)
Tensile Strength	80
Tensile Modulus	3300
Flexural Strength	125
Flexural Modulus	3200

**Table 4:** Coding of CFRP and GFRP samples.

**Fiber Type**	G (GFRP)	C (CFRP)
**Number of Layers**	7 (7 floors)	8 (8 floors)
**Environment**	Dry (K), Monthly residence times in sea water (1A, 2A, 3A, 6A, 15A)	Dry (K), Monthly residence times in sea water (1A, 2A, 3A, 6A, 15A)
**Sample No**	1, 2, 3	1, 2, 3

**Table 5:** Moisture retention rates (%) of GFRP specimens exposed to seawater for different durations.

Dry Sample Code	Dry Weight (g)	Store Time	Soaked in Sea Water Sample Code	Wet Weight (g)	Moisture Retention Rate (%)
G-7-K	17.2374	1st Month (720 h)	G-7-1A	17.3524	0.66
G-7-K	17.2374	2nd Month (1440 h)	G-7-2A	17.8593	3.43
G-7-K	17.2374	3rd Month (2160 h)	G-7-3A	17.9707	4.16
G-7-K	17.2374	6th Month (4320 h)	G-7-6A	18.0389	4.65
G-7-K	17.2374	15th Month (10,800 h)	G-7-15A	18.0699	4.83

**Table 6:** Moisture retention rates (%) of CFRP specimens exposed to seawater for different durations.

Dry Sample Code	Dry Weight (g)	Store Time	Soaked in Sea Water Sample Code	Wet Weight (g)	Moisture Retention Rate (%)
C-8-K	14.7695	1st Month (720 h)	C-8-1A	14.8808	0.57
C-8-K	14.7695	2nd Month (1440 h)	C-8-2A	14.9247	0.86
C-8-K	14.7695	3rd Month (2160 h)	C-8-3A	14.9252	0.87
C-8-K	14.7695	6th Month (4320 h)	C-8-6A	14.9326	0.92
C-8-K	14.7695	15th Month (10,800 h)	C-8-15A	14.9385	0.96

**Table 7:** Connection line damage conditions observed according to dry environment and sea water retention time.

Duration	Sample Type	Observed Damage Type	Damage Location
Dry environment	GFRP (G-7-K)	Adhesive bond line	Break in all samples
Dry environment	CFRP (C-8-K)	Adhesive bond line	Break in all samples
1 Month (720 h)	GFRP (G-7-1A)	Adhesive bond line	No Rupture in all samples
1 Month (720 h)	CFRP (C-8-1A)	Adhesive bond line	Sudden rupture (C-8-1A-1, C-8-1A-2) and Residual rupture (C-8-1A-3)
2 Months (1440 h)	GFRP (G-7-2A)	Adhesive bond line	No rupture (G-7-2A-1), rupture (G-7-2A-2, G-7-2A-3)
2 Months (1440 h)	CFRP (C-8-2A)	Adhesive bond line	Sudden rupture (C-8-2A-1) and Residual rupture (C-8-2A-2, C-8-2A-3)
3 Months (2160 h)	GFRP (G-7-3A)	Adhesive bond line	Residual rupture in all samples
3 Months (2160 h)	CFRP (C-8-3A)	Adhesive bond line	Sudden rupture (C-8-3A-1) and Residual rupture (C-8-3A-2, C-8-3A-3)
6 Months (4320 h)	GFRP (G-7-6A)	Deformations in the adhesive bond line	Residual rupture in all samples
6 Months (4320 h)	CFRP (C-8-6A)	Adhesive bond line	Residual rupture in all samples
15 Months (10,800 h)	GFRP (G-7-15A)	Deformations in the adhesive bond line	Sudden rupture in all samples
15 Months (10,800 h)	CFRP (C-8-15A)	Adhesive bond line	Residual rupture in all samples

**Table 8:** Young’s Modulus (E) of GFRP specimens.

Sample Code (GFRP)	E (GPa)	Evaluation
G-7-K	5.39	Dry environment (reference)
G-7-1A	5.07	% 5.94
G-7-2A	4.91	% 8.90
G-7-3A	4.69	% 12.98
G-7-6A	4.09	% 24.11
G-7-15A	4.03	% 25.23

**Table 9:** Young’s Modulus (E) of CFRP Specimens.

Sample Code (GFRP)	E (GPa)	Evaluation
C-8-K	11.50	Dry environment (reference)
C-8-1A	11.36	% 1.28
C-8-2A	11.11	% 3.39
C-8-3A	11.07	% 3.74
C-8-6A	10.44	% 9.21
C-8-15A	10.22	% 11.13

**Table 10:** Stress–strain values obtained from GFRP specimens and observed damage.

Sample Code	Stress (MPa)	Strain (ε)	Observed Damage (SEM)
G-7-K	63.0726	Min: 0.0128 Max: 0.0129	Damage onset, fiber fractures and breaks
G-7-1A	First: 72.2704 Min: 54.4886 Max: 80.0818	First: 0.0163 Min: 0.0113 Max: 0.0347	Fiber fractures, breaks, Irregularities and wears on matrix surface
G-7-2A	First: 75.8136 Min: 50.8163 Last: 75.6766	First: 0.0156 Min: 0.0094 Last: 0.0154	Fiber breakage, rupture, delamination
G-7-3A	75.6723	0.0146	Fiber breakage, rupture, fiber–matrix separation
G-7-6A	58.4044	0.0160	Matrix cracks, Separation at the fiber–matrix interface, delamination matrix plasticization (softening)
G-7-15A	First: 57.55439 Min: 50.4088 Last: 53.4619	First: 0.0159 Min: 0.0129 Last: 0.0140	Increased delamination, fiber removal and breakage, matrix dissolution, fiber–matrix separation advanced

**Table 11:** Stress–strain values obtained from CFRP specimens and observed damage.

Sample Code	Stress (MPa)	Strain (ε)	Observed Damage (SEM)
C-8-K	146.5976	0.0131	Initiation of damage: fiber fractures, no fiber–matrix separation
C-8-1A	First: 72.4395 Max: 83.6555	First: 0.0065 Max: 0.0077	Fiber breakage Slight irregularities and abrasions on the matrix surface
C-8-2A	126.2889	0.0112	Initial matrix cracks, interface separations Fiber breakage
C-8-3A	69.1586	0.0107	Gaps between fiber and matrix are evident, micropore formation is accelerated
C-8-6A	109.3453	0.0160	Fiber pull-outs, fiber breaks and ruptures
C-8-15A	112.2266	0.0110	Fiber breakage, porosity formation in internal areas and increase in micro voids

## Data Availability

The original contributions presented in the study are included in the article, further inquiries can be directed to the corresponding author.
